# Supplementary material for: Development of Anticancer Peptides Using Artificial Intelligence and Combinational Therapy for Cancer Therapeutics
Source: Pharmaceutics. 2022 May 6;14(5):997. doi: 10.3390/pharmaceutics14050997 (PMC9147327; doi:10.3390/pharmaceutics14050997)
Supplement: Supplementary file 1 [file pharmaceutics-14-00997-s001.zip › pharmaceutics-1679606-supplementary.pdf]

Table S1. List of peptide sequences predicted by AI

Sequence	Method	Treated cell	Inhibition index (μM)	Reference
GILSSIKGVAKGVAKNVAAQLLDTLKCKITGC	ML	A549,<br>MCF7,<br>HeLa,<br>LoVo,<br>293T	A549: 15.32 [IC_{50}],<br>MCF7: 45.25 [IC_{50}],<br>HeLa: 37.23 [IC_{50}]<br>LoVo: 59.78 [IC_{50}],<br>293T: 63.16 [IC_{50}]	
GIFDVVKGVLKGVGKNVAGSLLEQLKCKLSGGC	ML	A549,<br>MCF7,<br>HeLa,<br>LoVo,<br>293T	A549: 2.975 [IC_{50}],<br>MCF7: 25.74 [IC_{50}],<br>HeLa: 19.69 [IC_{50}]<br>LoVo: 8.05 [IC_{50}],<br>293T: 5.832 [IC_{50}]	
GVLGAVKDLLIGAGKSAAQSVLKTLSCKLSNDC	ML	A549,<br>MCF7,<br>HeLa,<br>LoVo,<br>293T	A549: 27.31 [IC_{50}],<br>MCF7: 41.21 [IC_{50}],<br>HeLa: 52.83 [IC_{50}]<br>LoVo: 55.22 [IC_{50}],<br>293T: 48.7 [IC_{50}]	**[1]**
GLFLDTLKGAAKDVAGKLEGLKCKIT	ML	A549,<br>MCF7,<br>HeLa,<br>LoVo,<br>293T	A549: 24.01 [IC_{50}],<br>MCF7: 37.84 [IC_{50}],<br>HeLa: 23.26 [IC_{50}]<br>LoVo: 35.05 [IC_{50}],<br>293T: 50.66 [IC_{50}]	
GLFDVVKGVLKGAGKNVAGSLLEQLKCK	ML	A549,<br>MCF7,<br>HeLa,<br>LoVo,<br>293T	A549: >128 [IC_{50}],<br>MCF7: >128 [IC_{50}],<br>HeLa: >128 [IC_{50}]<br>LoVo: 128 [IC_{50}],<br>293T: >128 [IC_{50}]	
RLLRLIRKLII	ML	MCF7,<br>A549,<br>LU1205,<br>Jurkat,<br>HDMEC	MCF7: 11 ± 1 [EC_{50}],<br>A549: 21 ± 1 [EC_{50}],<br>LU1205: 9 ± 1 [EC_{50}],<br>Jurkat: 39 ± 1 [EC_{50}],<br>HDMEC: 141 ± 1 [EC_{50}]	**[2]**
RLLRLIRKLIL	ML	MCF7,<br>A549,<br>LU1205,<br>Jurkat,<br>HDMEC	MCF7: 9 ± 1 [EC_{50}],<br>A549: 14 ± 1 [EC_{50}],<br>LU1205: 7 ± 1 [EC_{50}],<br>Jurkat: 25 ± 1 [EC_{50}],<br>HDMEC: 125-250 [EC_{50}]	

LLLLLIRKLIK	ML	MCF7,<br>A549,<br>LU1205,<br>Jurkat,<br>HDMEC	MCF7: 11 ± 1 [EC_{50}],<br>A549: 22 ± 1 [EC_{50}],<br>LU1205: 16 ± 1 [EC_{50}],<br>Jurkat: 25 ± 1 [EC_{50}],<br>HDMEC: 125-250 [EC_{50}]
YLLYLIRKLIL	ML	MCF7,<br>A549,<br>LU1205,<br>Jurkat,<br>HDMEC	MCF7: 13 ± 1 [EC_{50}],<br>A549: 29 ± 1 [EC_{50}],<br>LU1205: 14 ± 1 [EC_{50}],<br>Jurkat: 22 ± 1 [EC_{50}],<br>HDMEC: 256 ± 2 [EC_{50}]
RLLRLIRKLIR	ML	MCF7,<br>A549,<br>LU1205,<br>Jurkat,<br>HDMEC	MCF7: 14 ± 1 [EC_{50}],<br>A549: 27 ± 1 [EC_{50}],<br>LU1205: 16 ± 1 [EC_{50}],<br>Jurkat: 27 ± 1 [EC_{50}],<br>HDMEC: 138 ± 7 [EC_{50}]
QLLQLIRKLII	ML	MCF7,<br>A549,<br>LU1205,<br>Jurkat,<br>HDMEC	MCF7: 22 ± 1 [EC_{50}],<br>A549: 29 ± 1 [EC_{50}],<br>LU1205: 21 ± 1 [EC_{50}],<br>Jurkat: 35 ± 1 [EC_{50}],<br>HDMEC: 171 ± 1 [EC_{50}]
QLLQLIRKLIM	ML	MCF7,<br>A549,<br>LU1205,<br>Jurkat,<br>HDMEC	MCF7: 33 ± 1 [EC_{50}],<br>A549: 33 ± 1 [EC_{50}],<br>LU1205: 24 ± 1 [EC_{50}],<br>Jurkat: 46 ± 1 [EC_{50}],<br>HDMEC: 142 ± 2 [EC_{50}]
MLLMLIRKLIL	ML	MCF7,<br>A549,<br>LU1205,<br>Jurkat,<br>HDMEC	MCF7: 16 ± 1 [EC_{50}],<br>A549: 58 ± 1 [EC_{50}],<br>LU1205: 31 ± 1 [EC_{50}],<br>Jurkat: 31 ± 1 [EC_{50}],<br>HDMEC: 262 ± 1 [EC_{50}]
QLLQLIRKLIR	ML	MCF7,<br>A549,<br>LU1205,<br>Jurkat,<br>HDMEC	MCF7: 53 ± 1 [EC_{50}],<br>A549: 60 ± 1 [EC_{50}],<br>LU1205: 38 ± 1 [EC_{50}],<br>Jurkat: 75 ± 1 [EC_{50}],<br>HDMEC: 260 ± 1 [EC_{50}]
QLLQLIRKLIK	ML	MCF7,<br>A549,	MCF7: 50-100 [EC_{50}],<br>A549: 54 ± 1 [EC_{50}],

		LU1205,<br>Jurkat,<br>HDMEC	LU1205: 39 ± 1 [EC_{50}],<br>Jurkat: 69 ± 1 [EC_{50}],<br>HDMEC: ≈250 [EC_{50}]
QLLQLIRKLIY	ML	MCF7,<br>A549,<br>LU1205,<br>Jurkat,<br>HDMEC	MCF7: 50-100 [EC_{50}],<br>A549: 61 ± 1 [EC_{50}],<br>LU1205: 29 ± 1 [EC_{50}],<br>Jurkat: 58 ± 1 [EC_{50}],<br>HDMEC: 349 ± 1 [EC_{50}]
LLLQLIRKLIL	ML	MCF7,<br>A549,<br>LU1205,<br>Jurkat,<br>HDMEC	MCF7: 10 ± 1 [EC_{50}],<br>A549: 30 ± 1 [EC_{50}],<br>LU1205: 12 ± 1 [EC_{50}],<br>Jurkat: 53 ± 1 [EC_{50}],<br>HDMEC: 154 ± 1 [EC_{50}]
TLLLLIRKLIL	ML	MCF7,<br>A549,<br>LU1205,<br>Jurkat,<br>HDMEC	MCF7: 38 ± 1 [EC_{50}],<br>A549: 79 ± 1 [EC_{50}],<br>LU1205: 16 ± 1 [EC_{50}],<br>Jurkat: 30 ± 1 [EC_{50}],<br>HDMEC: 348 ± 1 [EC_{50}]
RLLLLIRKLIL	ML	MCF7,<br>A549,<br>LU1205,<br>Jurkat,<br>HDMEC	MCF7: 24 ± 1 [EC_{50}],<br>A549: 35 ± 1 [EC_{50}],<br>LU1205: 18 ± 1 [EC_{50}],<br>Jurkat: 40 ± 1 [EC_{50}],<br>HDMEC: 160 ± 1 [EC_{50}]
LLLLLIRKLIL	ML	MCF7,<br>A549,<br>LU1205,<br>Jurkat,<br>HDMEC	MCF7: 25 ± 1 [EC_{50}],<br>A549: >100 [EC_{50}],<br>LU1205: 48 ± 1 [EC_{50}],<br>Jurkat: 50 ± 1 [EC_{50}],<br>HDMEC: 278 ± 1 [EC_{50}]
QLLLLIRKLIL	ML	MCF7,<br>A549,<br>LU1205,<br>Jurkat,<br>HDMEC	MCF7: 63 ± 1 [EC_{50}],<br>A549: >100 [EC_{50}],<br>LU1205: 44 ± 1 [EC_{50}],<br>Jurkat: 39 ± 1 [EC_{50}],<br>HDMEC: 271 ± 1 [EC_{50}]
QLLLLIRKLIV	ML	MCF7,<br>A549,<br>LU1205,<br>Jurkat,	MCF7: 77 ± 1 [EC_{50}],<br>A549: >100 [EC_{50}],<br>LU1205: 53 ± 1 [EC_{50}],<br>Jurkat: >100 [EC_{50}],

		HDMEC	HDMEC: 339 ± 1 [EC_{50}]	
CLLLLIRKLIL	ML	MCF7,<br>A549,<br>LU1205,<br>Jurkat,<br>HDMEC	MCF7: >100 [EC_{50}],<br>A549: >100 [EC_{50}],<br>LU1205: 76 ± 1 [EC_{50}],<br>Jurkat: >100 [EC_{50}],<br>HDMEC: ≈500 [EC_{50}]	
KLWKKIEKLIKKLLTSIR	DL	MCF7	47 ± 3 [EC_{50}],<br>236 ± 13 [HC_{50}],<br>5.1 ± 0.6 [TI]	
YIWARAERVWLWWGKFLSL	DL	MCF7	56 ± 3 [EC_{50}],<br>inactive [HC_{50}],<br>>7 [TI]	
ELAKKLTKLKRQLHRIW	DL	MCF7	inactive [EC_{50}],<br>inactive [HC_{50}],<br>n.d. [TI]	
DLFKQLQRLFLGILYCLYKIW	DL	MCF7	47 ± 4 [EC_{50}],<br>132 ± 16 [HC_{50}],<br>2.8 ± 0.6 [TI]	
KLIDQWKKVLYHVE	DL	MCF7	inactive [EC_{50}],<br>inactive [HC_{50}],<br>n.d. [TI]	**[3]**
AIKKFGPLAKIVAKV	DL	MCF7	95 ± 4 [EC_{50}],<br>inactive [HC_{50}],<br>>4 [TI]	
RWNGRIIKGFYNLVKIWKDLKG	DL	MCF7	42 ± 4 [EC_{50}],<br>89 ± 6 [HC_{50}],<br>2.1 ± 03 [TI]	
KVWKIKKNIRRLLHGIKRGWKG	DL	MCF7	34 ± 4 [EC_{50}],<br>inactive [HC_{50}],<br>>11 [TI]	
GFWARIGKVFAAVKNL	DL	MCF7	101 ± 4 [EC_{50}],<br>inactive [HC_{50}],<br>>4 [TI]	

AFLYRLTRQIRPWWRWLYKW	DL	MCF7	45.5 ± 0.8 [EC_{50}], 34 ± 5 [HC_{50}], 0.7 ± 0.1 [TI]	
RIWGKHSRYIKIVKRLIQ	DL	MCF7	50 ± 10 [EC_{50}], inactive [HC_{50}], >8 [TI]	
QIWHKIRKLWQIIKDGF	DL	MCF7	16.1 ± 0.3 [EC_{50}], 23 ± 5 [HC_{50}], 1.4 ± 0.3 [TI]	
ALFGILKKAFGKILTIFAGLPGVV	ML	MCF7, A549	MCF7: 9.8 [EC_{50}], A549: 8.6 [EC_{50}]	
GLGDFIKAIAKHLGPLIGILPSKLKVAA	ML	MCF7, A549	MCF7: 4.5 [EC_{50}], A549: 11.3 [EC_{50}]	
GLKDPLKSVAKHLVK	ML	MCF7, A549	MCF7: >100 [EC_{50}], A549: >100 [EC_{50}]	
GLVMLVKKAGLT	ML	MCF7, A549	MCF7: >100 [EC_{50}], A549 >100: [EC_{50}]	
IIFGVIKALSG	ML	MCF7, A549	MCF7: >100 [EC_{50}], A549: >100 [EC_{50}]	
FLGPTIGKIAKFILKHIVGLGDAALV	ML	MCF7, A549	MCF7: 2.6 [EC_{50}], A549: 10.7 [EC_{50}]	**[4]**
GIVTILKGAVGVILGGL	ML	MCF7, A549	MCF7: >100 [EC_{50}], A549: >100 [EC_{50}]	
GLFKIISGIA	ML	MCF7, A549	MCF7: >100 [EC_{50}], A549: >100 [EC_{50}]	
GLGMIAHGV	ML	MCF7, A549	MCF7: >100 [EC_{50}], A549: >100 [EC_{50}]	
GLWKILKKGA	ML	MCF7, A549	MCF7: >100 [EC_{50}], A549: >100 [EC_{50}]	
GLFAILKKLVNLVG	ML	MCF7, A549	MCF7: 2.3 [EC_{50}], A549: 4.6 [EC_{50}]	

GLFKIISKLAKKA	ML	MCF7, A549	MCF7: 27.7 [EC_{50}], A549: 36.3 [EC_{50}]
GLPLFLKKLGSAV	ML	MCF7, A549	MCF7: >100 [EC_{50}], A549: >100 [EC_{50}]
GLWGVIKALGGHVG	ML	MCF7, A549	MCF7: >100 [EC_{50}], A549: 69.3 [EC_{50}]
GLFLVALKQVATIKKLIGGLFAVVGVA	ML	MCF7, A549	Synthesis failed

IC_{50}: Half-inhibitory concentration, EC_{50}: half-effective concentration, HC_{50}: half-hemolytic concentration, TI: Therapeutic index (HC_{50}/ EC_{50}), AI: Artificial intelligence, n.d.: no data, ML: Machine learning, DL: Deep learning

References

1. Zhao, Y.; Wang, S.; Fei, W.; Feng, Y.; Shen, L.; Yang, X.; Wang, M.; Wu, M. Prediction of Anticancer Peptides with High Efficacy and Low Toxicity by Hybrid Model Based on 3D Structure of Peptides. *Int. J. Mol. Sci.* **2021**, *22*, 5630.
2. Grisoni, F.; Neuhaus, C.S.; Gabernet, G.; Müller, A.T.; Hiss, J.A.; Schneider, G. Designing anticancer peptides by constructive machine learning. *ChemMedChem*. **2018**, *13*, 1300-1302.
3. Grisoni, F.; Neuhaus, C.S.; Hishinuma, M.; Gabernet, G.; Hiss, J.A.; Kotera, M.; Schneider, G. De novo design of anticancer peptides by ensemble artificial neural networks. *J. Mol. Model*. **2019**, *25*, 112.
4. Lin, Y.C.; Lim, Y.F.; Russo, E.; Schneider, P.; Bolliger, L.; Edenharter, A.; Altmann, K.H.; Halin, C.; Hiss, J.A.; Schneider, G. Multidimensional design of anticancer peptides. *Angew. Chem. Int. Ed*. **2015**, *54*, 10370-10374.
